# Ilizarov Ring Fixator in the Lower Limb for 2000 Days: A Case Report

**DOI:** 10.7759/cureus.43891

**Published:** 2023-08-21

**Authors:** Nareshkumar Dhaniwala, Shivshankar Jadhav, Aditya Chirayath, Amit Saoji

**Affiliations:** 1 Department of Orthopaedic Surgery, Datta Meghe Institute of Higher Education and Research, Wardha, IND

**Keywords:** infection, distraction histogenesis, deformity, non-union, ilizarov

## Abstract

Ring external fixators developed by Gavriil Abramovich Ilizarov from Russia are used to treat the difficult cases of infected non-union, shortening of limbs by bone lengthening, and deformity correction in joints and bones in isolation or in combination. Fixation of the involved bone with the ring is commonly achieved using four rings, each having two Ilizarov wires that are passed along the superior surface of the ring, then across the bone, and exiting out on the opposite side touching the surface of the ring. The case report herein reports a patient who kept the ring fixator on his thigh without any problem for a long period of five and a half years. The case is being reported due to the abnormally long period of ring fixator application without any complications and the excellent result achieved in terms of union and infection control.

## Introduction

Professor Gavriil Abramovich Ilizarov (1921-1992) from Russia introduced a new type of circular/ring external fixator in the 1950s. Ring external fixators are used to treat the difficult cases of infected non-union, shortening of limbs by bone lengthening, and deformity correction in joints and bones in isolation or in combination [1]. The basic principles used in ring fixators are distraction neo-osteogenesis, compression at the delayed union or non-union site, acute compression, and bone lengthening.

Normally, the ring fixator assembly consists of a minimum of three or four rings interconnected with connecting rods. A full ring is made up of two half rings connected with each other by ring fixation bolts and nuts. Fixation of the involved bone with the ring is commonly achieved using two Ilizarov wires, which are passed along the superior surface of the ring, then across the bone, and exiting out on the opposite side touching the surface of the ring. Each wire is fixed to the ring using wire fixation bolts and nuts. After tightening the wire on one side, tension is applied to the wire and the opposite side nuts are tightened. Two tensioned wires through the bone and attached to the ring provide stability to the bone and make full weight-bearing possible. All the rings are similarly fixed using specialized wires passing across the involved bone. Olive wires provide an important buttress effect in the correction of angular deformity. In the femur, Schanz screws/pins are used many times to provide better stability. It is commonly used in the proximal femur where full ring application is impractical. In a ring fixator assembly, the proximal-most and distal-most rings are typically applied at the upper and lower metaphyseal parts of the long bone. The other two rings’ position depends on the site of the fracture or non-union, corticotomy, or deformity. One ring is applied proximal to the disease site and the other distal to it. All the rings are connected to each other by threaded connecting rods, at least three to four in number, thus permitting distraction or compression between rings. This versatile facility permits compression, distraction, and alternate compression-distraction and helps to achieve the desired result. Osteogenesis depends on the stability of the external frame and is dependent on bony stability. Ring characteristics have a significant influence on frame stability; rings with a big diameter are less stable than rings with a smaller diameter [2]. By application of a distractor at the suitable site, ring fixators have been used for gradual deformity correction successfully. The duration of the fixator use depends on the purpose and progress achieved in the treatment goal. On average, the ring fixator may be used for a duration of three to six months or more. In cases of limb length correction, it might be used for a longer period extending to one year or more [3-5]. The proper application of the Ilizarov ring fixator requires meticulous care at each step.

Infection at the site of entry of the wire in the bone and reduction in the tension of the wires are two common complications seen while using ring fixation in long bones [2]. Regular care of the wire entry site by cleaning and dressing with a betadine-soaked small gauze piece is required to prevent infection. The wires may need to be tensioned after a few weeks. The patients commonly complain of pain on weight-bearing in case of any of these complications. The case report herein reports a patient who kept the ring fixator on his thigh without any problem for a long period of five and a half years. The case is being reported due to the abnormally long period of ring fixator application without any complications and the excellent result achieved in terms of union and infection control.

## Case presentation

A 15-year-old boy, a resident of the rural region of Maharashtra in central India, sustained an open fracture of the left thigh bone and crush injuries in both feet in a road traffic accident in March 2017. He reported to the regional tertiary care health center after a delay of two days and was treated by mid-right foot amputation, debridement, Kirschner wire fixation of metatarsals in the left foot, and external fixation of the left thigh fracture. There was extensive loss of skin on the left thigh, which was managed with split-thickness skin grafting in stages from the opposite thigh. The patient had developed multiple discharging wounds in the left thigh within a few days of fixation and remained bedridden as he was unable to stand on his own owing to the morbidity of both feet and both thighs. He was discharged from the hospital with the advice of dressing wound sites, continuing the external fixator, and resting for an unspecified length of time. As the discharge from the left thigh wounds was persistent and not responding to drugs and a good diet, the patient reported to our center almost eight months after the initial trauma.

**Figure 1:**
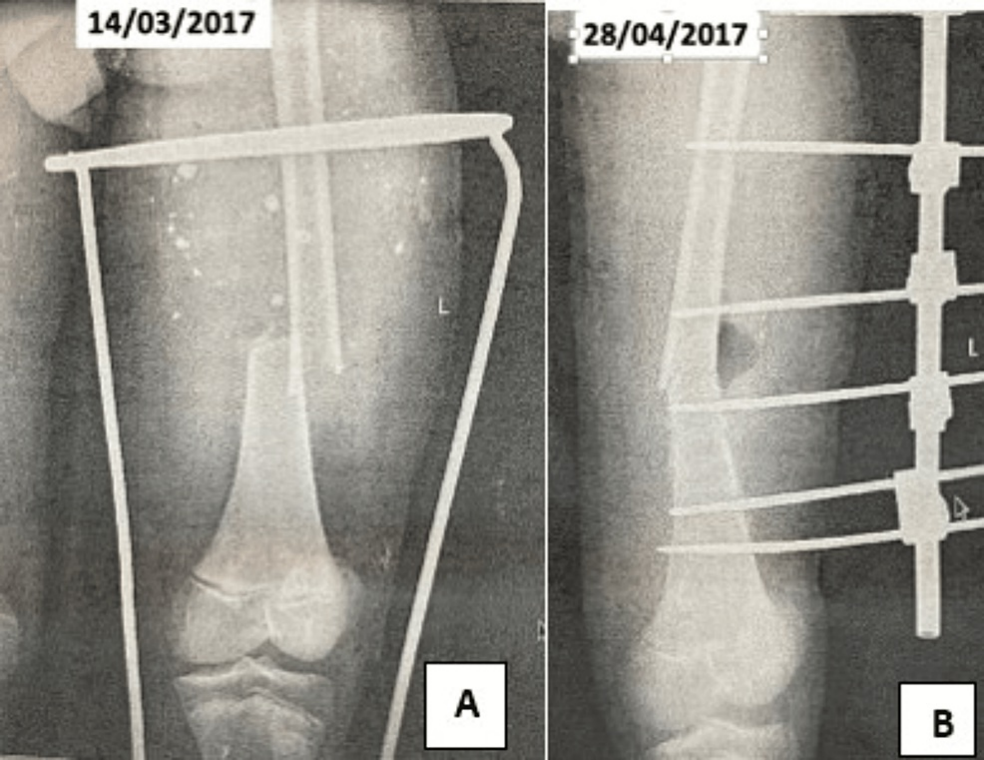
X-ray shows (A) a lower third shaft femur fracture and (B) a unilateral external fixator with angulation at the fracture site.

A physical examination in November 2017 demonstrated multiple active discharging sinuses from the front and back of the left thigh along with a unilateral uniplanar external fixator applied to the thigh. There was evidence of skin grafting on the left thigh anteromedial aspect. The dorsum of the left foot distal part was scarred, and the toes and ankle had restricted mobility with mild equinus deformity at the left ankle. The right foot was amputated at the tarsometatarsal level, and the stump had healed in the equinovarus position. The knee range of movement was 0-30° on the left side and 0-90° on the right. The patient had not ambulated since the accident. X-rays of the left thigh at the time of injury and one month after external fixation showed a transverse fracture at the distal third femur stabilized with unilateral Schanz pins, connecting rod with AO clamps (Figure 1A, B). As there was continuous infection and the pins were loose, the fixator was removed and the wounds were dressed after regular irrigation of the tract with betadine mixed with normal saline. The patient was put on sensitive antibiotics in combination intravenously, and the infection was brought under control.

**Figure 2:**
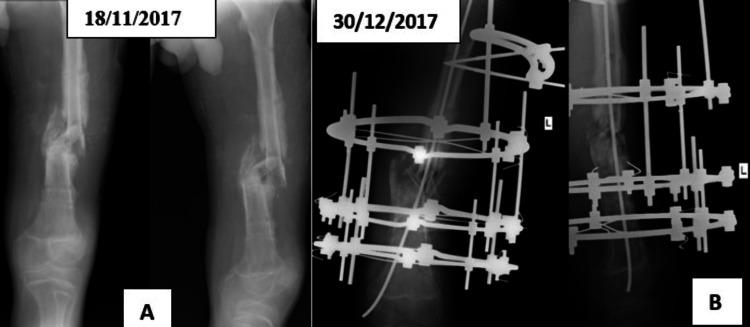
(A) Anteroposterior and lateral X-rays show a non-united fracture. (B) X-ray after fixation with ilizarov ring fixator as well as TENS nail in situ. TENS: Titanium Elastic Nailing System.

An X-ray of the thigh showed frank non-union without any bridging callus formation, a gap at the fracture site, and evidence of persistent infection in the form of an ischemic segment at the fracture site and poor quality new bone formation in both fragments (Figure 2A). In view of this, it was planned to stabilize the femur with Ilizarov ring fixator. The ring fixator application was done on November 22, 2017. The ring fixator consisted of one femoral arc with two Schanz pins and one full ring made of two half rings with two tensioned wires in the proximal fragment. The distal fragment was fixed with two full rings, each made of two half rings and each having two tensioned wires. The rings were connected using proper connecting rods and nuts, and the fixator assembly provided sufficient stability after tensioning the wires passed through the safe zones. The care of wires was done regularly and was taught to the patient as well. The post-fixation X-ray showed the fracture ends just in contact after the ring fixator application in the AP view, and a gap was seen between them in the lateral view. The fixator was realigned on December 30, 2017, improving the fracture ends contact (Figure 2B). Simultaneously, the ipsilateral fibula segment was harvested and placed as a bone graft at the gap, and a single intramedullary titanium elastic nail was passed from the condyle upwards across the non-union till trochanteric region to guide callus formation and help union. The patient was discharged on February 22, 2018, with supportive drugs, advice regarding the care of pin tracts, exercises for both knees and both ankles, and instructions to use a walker for walking support. He was advised to follow up after one month. He had minimal serous discharge from a sinus on his left thigh at the time of discharge.

**Figure 3:**
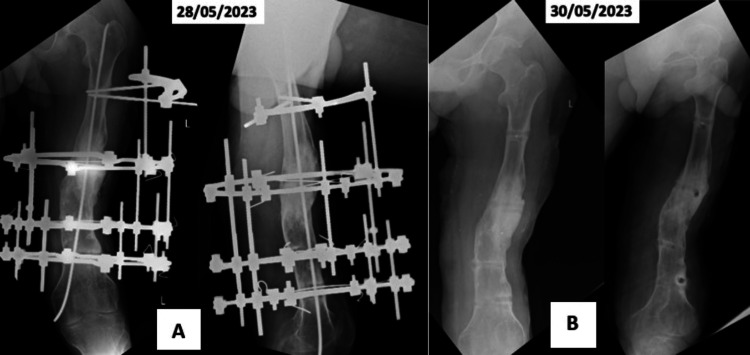
(A) X-ray shows a united and remodeled non-union site with slight angulation, ring fixator assembly, and TENS nail without any osteolysis. (B) Post-fixator removal X-ray showing tracts and evidence of healed infection.

The patient never turned up for follow-up and remained at home, taking personal care of the fixator wires and sinus site. The discharging wound healed gradually in about a year since his discharge from the hospital. Later on, he avoided hospital visits due to the COVID-19 epidemic in 2020 and its long-lasting scare in society in 2021 and 2022. He never attempted to stand on his own and was carrying on with his life with the ring fixator assembly in place. The right foot stump gradually became more bent. He was seen by a social worker in a health camp in his area and was persuaded to visit the hospital. He reported to us again on May 28, 2023, more than five and a half years or 2013 days after the ring fixator application. Examination revealed the ring fixator assembly along with all its components well in place. There was no discharge from any wire or pin site. All the wires, rings, connecting rods, and fixation bolts were in the proper position and tightness. The X-ray showed the fracture to be well united and remodeled with an intramedullary single Titanium Elastic Nailing System (TENS) in place. The fixator assembly and the TENS nail were easily removed on May 30, 2023 (Figure 3A, B). The fracture was mal-united clinically and radiologically. The X-ray taken after the removal of the fixator and TENS shows the tract of wires, holes of probably previous unilateral external fixator or tracts after healed infection in the bone, and posteromedial angulation at the united fracture site. The fibular graft was well incorporated into the remodeled callus. 

As the patient was not able to put the amputated stump on the ground due to equinus deformity, it was freshened by removing a wedge-shaped extra bone. The heel pad fat and calcaneus were retained for smooth weight transmission. The patient was taught knee and ankle mobilization exercises and made to stand with the support of a walker. He is under follow-up for rehabilitation regarding ambulation and joint exercises.

## Discussion

Professor Gavriil Ilizarov (1921-1992) introduced a new type of circular/ring external fixator in the 1950s. He developed the concept of tension stress for the direction and control of tissue growth, thus indicating the new doctrine of tissue generation under the influence of bone distraction. He noted that normally new bone columns appear in three to four weeks after gradual distraction at a rate of 1 mm divided in fractions 6/8 hourly every day. The new bone commonly becomes defined in six to eight weeks. But the circular fixator needs to be retained for at least twice as longer as the distraction period for bone maturity. The clear X-ray appearance of the regenerated calcified bone with its canalization marks the time of circular fixator removal [1]. During the treatment phase using a ring fixator, repeated good quality X-rays need to be taken at frequent intervals to assess the formation of the callus at the fracture site and regenerate at the corticotomy site. Treatment is continued till a satisfactory amount of callus or regeneration is seen. Time equal to or double the time elapsed for callus formation is permitted for consolidation and remodeling of callus at the involved site. After this decision regarding the removal of the fixator is taken, tension is reduced in the wires and the patient is permitted to ambulate as earlier. If there is no pain, the fixator can be removed by cutting the wires and loosening nuts and bolts. Finally, wire tracts are irrigated with saline and povidone-iodine, and dressing is done as per need.

On average, the fixators are kept for three to six months. In limb lengthening cases, many times frames are needed for longer periods depending on the amount of distraction needed and the quality of bone formation. A longer period predisposes to pin tract infection and a release in the tension of the wires.

Complications due to the use of the Ilizarov circular fixator may be general, due to the method; specific, related to the technique; and inflammatory [1]. General complications are detected early and include neural/vascular penetration by the wires, comminution, and displacement of fractured or osteotomized bone. Pain, compartment syndrome, local edema, muscle contracture, and hypertension are a few other general complications presenting within a few days or weeks. Specific complications related to technique include local swelling, skin tightness, pain on movement, bending or breakage of wire, deviation of displaced fragment, joint stiffness, minimal regeneration, fusion of corticotomy site, and sometimes psychological disturbance to the patient. Posterior subluxation of the tibia and equinus at the ankle are two commonly noted complications. Pin tract infection, osteomyelitis, and phlebitis are inflammatory complications.

Ilizarov distraction osteogenesis has been noted to be an effective modality for achieving union in slow and non-uniting fracture cases; it also helps in the correction of limb length discrepancies if there is any shortening due to bone loss. It has high patient compliance, easy wound care, and low rate of complications such as infection, deformity, and shortening. Ajmera et al. noted the mean time for Limb Reconstruction System as 44 weeks, with a range of 24-51 weeks [3]. Giannoudis et al. in their study on 169 distal tibial fractures by circular external fixator noted the median time for union to be 166.5 days, the range being 104-537 days [4]. Another study related to limb lengthening had the time range for fixator varying from four to 16 months [5]. In a study by Subasi et al. done on 15 open comminuted tibial condyle fractures using circular external fixation frames, union time was 22.8 weeks (range 16-44 weeks) [6]. The median duration of the Ilizarov external fixator (IEF) was noted to be 174 days (56-614 days) in a study done on 89 patients to assess patients’ lives post-Ilizarov external fixation [7]. In a study of Ilizarov fixator in high-energy pilon fractures, the mean time of healing was noted to be 15.8 weeks (13-23 weeks) [8]. Pin tract infection was noted in nine out of 15 cases, but no one had a deep infection or osteomyelitis. In another study done on 56 patients with complex tibial fractures, the ring fixator average removal time was 25.3 weeks (range 9-53 weeks). A circular external fixator has been successfully used as a definitive treatment for open and comminuted femoral fractures and tibial-infected non-union cases [9,10]. Ilizarov fixation has also been used for non-free bone plasty in the management of long bone defects and non-union [11].

In our case, the patient had a circular external fixator on his left thigh from November 2017 to April 2023, a duration of 5.5 years, or 66 months, or 2007 days precisely. During this extended period of fixator on the limb, he did not face any notable complications. He remained in follow-up for only a month after the fixator application. His fixator was realigned, and a TENS nail was passed negotiating through the fracture site within this period only. After that, due to COVID-19 and personal reasons, the patient never turned up at the hospital. His infection gradually got controlled, and the fracture proceeded through union, ultimately consolidating into a strong bone. Such incidences are not common and have not been reported in the literature.

## Conclusions

Ilizarov ring fixator, after proper application, commonly leads to the union of fracture or non-union site without any complications, despite the assembly remaining on the limb for a very long time. In open or comminuted femur fractures, circular external fixation can result in acceptable and consistent union rates and good to exceptional functional outcomes.
